# A Novel Approach to Vibro-Acoustic Fault Diagnosis Using Intrinsic Multidimensional Gnome Entropy Analysis and a Convolutional Neural Network

**DOI:** 10.3390/e28101133

**Published:** 2026-10-15

**Authors:** Hai Xu, Yanan Zhou, Haoxiang Liu, Qi Yuan

**Affiliations:** 1 School of Low-Altitude and Aerospace Equipment, Wuhan Technical College of Communications, Wuhan 430065, China; xuhai@whtcc.edu.cn; 2 Key Laboratory of Metallurgical Equipment and Control Technology, Ministry of Education, Wuhan University of Science and Technology, Wuhan 430081, China; liuhaoxiang@wust.edu.cn; 3 Hubei Key Laboratory of Mechanical Transmission and Manufacturing Engineering, Wuhan University of Science and Technology, Wuhan 430081, China; 4 School of Automotive Intelligent Manufacturing, Hubei University of Automotive Technology, Shiyan 442002, China; 202401444@huat.edu.cn

**Keywords:** fault diagnosis, vibro-acoustic signals, variational mode decomposition, band-limited intrinsic mode functions, multidimensional mean Gnome entropy, convolutional neural network

## Abstract

Due to the complex practical working conditions, fault diagnosis is very challenging in rotary machinery. Rotary machinery is often used in places with a high temperature, strong corrosion, or where acceleration sensors are hard to install. Thus, acoustical signal processing becomes a powerful tool for diagnosing faults in rotary machinery. This paper proposes a novel strategy called intrinsic multidimensional Gnome entropy (IMGE) analysis that can extract the quantitative features of vibro-acoustic signals. The method consists of signal analysis using variational mode decomposition (VMD), and feature extraction using the proposed mean Gnome entropy (MGE). Multidimensional MGE solves the issue of negative computation values in the original algorithm and improves the accuracy and stability of entropy in multiple dimensions. VMD is used to decompose vibro-acoustic signals into a series of band-limited intrinsic mode functions (BLIMFs) denoting the dynamical characteristics of signals. Multidimensional MGE values of BLIMFs are extracted as quantitative features and inputted into a convolutional neural network (CNN) to diagnose faults in rotary machinery. The improved performance of the proposed framework under the tested experimental conditions is verified by theoretical derivation, numerical simulations, and research on practical applications.

## 1. Introduction

Rotary machinery is very common in mechanical, civil, and aerospace engineering, and diagnosing its faults plays a vital role in ensuring safe and stable operation [1,2]. Previous studies on this topic have mainly focused on extracting and analyzing the signal’s time and frequency domain characteristics to diagnose mechanical faults. The nonlinear and nonstationary vibrations caused by impacts, sudden speed changes, structural deformation, and friction changes make mechanical fault diagnosis very difficult [3,4]. The feature extraction of vibration signals is usually suitable for diagnosing faulty parts; however, this can be difficult under certain conditions. These situations require a novel and effective way to accurately diagnose faults.

Physically, vibration causes an acoustical signal. Although the dynamical characteristics of the vibration and acoustical signals are different, they both contain information on the equipment’s status. Acoustical signal processing has unique advantages over vibration signal processing. When the equipment is operating under high temperatures and strong corrosion, or where the acceleration sensor is hard to install, acoustical signal processing becomes a useful tool to diagnose mechanical faults [5]. Mechanical fault diagnosis be analyzed by processing acoustic signals and by experiences in industrial scenarios. When a gear has a defect, the meshing sound is piercing, and so the fault can be identified by an experienced expert. However, fault diagnoses relying on experience alone are inevitably inaccurate. Vibro-acoustic signal processing is a specialized non-contact method for fault diagnosis [6,7]. Vibro-acoustic signals also contain fault information, offering a non-contact and non-destructive strategy to diagnosing mechanical faults. Hartono et al. [8] conducted a study using non-contact acoustic sensors instead of vibration sensors to detect gearbox faults. They identified characteristic frequencies in acoustical signals, thus providing an alternative solution for mechanical fault diagnosis. Li et al. [9] proposed a deep learning fusion-based method by using acoustic and vibration signals to diagnose faults in a gearbox. The results show that this strategy improves diagnosis accuracy. Elasha et al. [10] used vibration and acoustic emission (AE) signal processing to diagnose the incipient fault of a planetary gearbox, with the method showing an advantage in identifying early defects. Bhuiyan et al. [11] used the AE technique to identify the growth of fatigue cracks. The waveform analysis of the in situ fatigue growth verifies its validity for monitoring structural health conditions. Vanraj et al. [12,13] proposed a non-contact fault diagnosis method involving processing the acoustic signal to extract fault features.

Feature extraction is an important part of acoustic signal processing: a failed feature extraction leads to inaccurate fault diagnosis. However, feature extraction of the acoustical signal is difficult under complex conditions. Traditional signal processing algorithms, such as wavelet packet decomposition [14], local mean decomposition [15], empirical mode decomposition (EMD) [16], and neural networks [17], are widely used. Of these, EMD is a powerful method that decomposes the signal into a series of intrinsic mode functions (IMFs) based on time scales, with complete and quasi-orthogonal properties. IMFs align in the order of high-to-low instantaneous frequencies. EMD does not require a pre-set basis and conducts decomposition adaptively. It has been applied to the fields of EEG signal processing [18,19], fault detection [20,21], and modal identification [22]. However, EMD has limitations, such as a lack of mathematical basis and a mode mixing problem, and so Dragomiretskiy et al. [23] proposed variational mode decomposition (VMD) as an optimized version of EMD. It uses a non-recursive variational strategy to extract band-limited intrinsic mode functions (BLIMF). The built-in Wiener filter makes the algorithm robust to the noises and sampling methods. It has good time-frequency resolution and a strict mathematical basis. Many studies have been carried out using VMD: Wang et al. [24] conducted a systematic study of VMD in oscillation extraction, detrend analysis, and impact detection; Hong et al. [25] adopted VMD to assess the degradation state of power transformer windings; Li et al. [26] adopted VMD to extract fault features of a planetary gearbox; and Yang et al. [27] embedded BLIMFs to high-dimensional phase space to extract fault features of rolling bearing. VMD has been used in fault diagnosis of rotary machinery [28], acoustic signal processing [29], and power prediction [25]. These studies show that it is efficient at extracting the dynamic characteristics of nonlinear and nonstationary signals.

Studies of VMD comprise two main aspects: signal preprocessing and parameter optimization. Peng et al. [30] proposed using warped VMD to process the signal by converting it into a phase-regulated warping signal, then conducting inverse warping transformation. Wen et al. [31] proposed fractional iterative VMD to map the signal into a fractal domain by using fractal Fourier transform to improve the decomposition performance. Parameter optimization improves the decomposition performance: Lin et al. [32] proposed sparsity-oriented VMD to select mode order using sparse optimization, thus improving the decomposition, and validated the method’s effectiveness by conducting fault diagnosis studies on a gearbox. Zhang et al. [33,34] proposed optimized VMD and generalized VMD, which optimize performance by using parameter optimization and information such as spectral position and bandwidth, which have been used in slip detection and feature extraction for sandwich cylindrical structures. Zhang et al. [35] proposed adaptive VMD to select important parameters using a global optimized algorithm. Zheng et al. [36] proposed dichotomy VMD, in which center frequencies are determined by the dichotomization method. In addition, the envelop nesting algorithm [37] and multi-criteria objective function [38] have also been applied to parameter optimization to improve the performance of VMD.

After the decomposition of the vibro-acoustic signals, the quantitative features are extracted to characterize the signal’s dynamic characteristics. Entropy is a powerful way to quantify the complexity and regularity of the signal [39]. Entropy includes the approximate entropy (AE) [40], sample entropy (SE) [41], permutation entropy [42], and their multiscale derivations [43,44]. AE measures the signal’s complexity and statistical quantification, and has been used to characterize the severity of defects [45,46]. SE was proposed as an improved version of AE that is robust to noise and can obtain stable entropy values from the short data [41]. SE performs self-matching and measures the new information with the signal itself, although it does occasionally produce false information. Multiscale sample entropy (MSE) is used to analyze the signal at multiple scales using coarse graining. It enriches the SE and reflects the signal’s dynamical characteristics [43]. Permutation entropy [44] introduced the idea of permutation for computing the complexity between the reconstructed subsequences, and has been used in fault diagnosis [47]. Multiscale entropy theories have been used in biomedical signal processing [48], pattern recognition [49], and fault diagnosis in rotary machinery [43]. However, entropy inevitably depends on parameters, including embedding dimension and scale factor; these parameters greatly affect the computation of entropy, and thus rank entropy (RE) was proposed [50]. Recently, Manis et al. [51] proposed bubble entropy (BE) to eliminate the scale factor. BE is derived from PE, in which the embedded subsequences are ranked according to a certain criterion. Bubble sort is used to order and count instead of conducing swaps for all sequences. A more coarse-grained distribution is obtained without the definition of scale factor. Inspired by the idea of BE, Wang et al. [52] proposed Gnome entropy (GE) by using Gnome sort instead of Bubble sort in ascending order. GE has better anti-noise ability and further solves the problem of parameter selection in quantifying the signal’s regularity, and it has been used to monitor bolt looseness.

The rest of the paper is presented as follows: Section 2 introduces the methodologies of VMD, mean Gnome entropy (MGE), and convolutional neural network (CNN). Section 3 elaborates the proposed fault diagnosis approach via intrinsic multidimensional Gnome entropy (IMGE) analysis and CNN by giving a detailed description and graphic illustration. Section 4 presents a numerical simulation of VMD to validate its superiority over GE, and validates the effect of MGE using the Case Western Reserve University bearing dataset. Section 5 validates the proposed method via the processing of vibro-acoustic signals of faulty gears and bearings. Section 6 concludes the paper and presents its major findings.

The main contribution is the proposed IMGE framework, in which VMD is used to obtain BLIMFs, multidimensional MGE is used to characterize the complexity and regularity of each mode, and the feature matrix is used as the input of a CNN classifier. Compared with conventional GE, the proposed MGE introduces amplitude-based Heaviside processing and mean aggregation across multiple dimensions. These operations are intended to reduce the influence of low-amplitude components, avoid abnormal negative entropy values, and improve the stability of multidimensional entropy features. This strategy leads to feature representation and processing of vibro-acoustic signals.

## 2. Methodology

### 2.1. Variational Mode Decomposition

VMD non-recursively decomposes a multi-component signal into a series of BLIMFs [23]. Its methodology is based on Wiener filtering, one-dimensional Hilbert transformation, and heterodyne demodulation. *K* order modes *u_k_* have specific sparse properties and limited bandwidths in the spectral domain. A smaller *K* causes a mode mixing problem, while a larger *K* causes redundant modes. Each mode has its center frequency *ω_k_*, and the bandwidth is estimated using *H*^1^ Gaussian smoothness. The variational model of VMD is as follows:

(1) $$\begin{array}{l} \underset{\left\{u_{k}\right\},\left\{\omega_{k}\right\}}{\min}\left\{\sum_{k}{\left\|\partial_{t}\left[\left(\delta (t)+\frac{j}{\mathrm{\pi t}}\right)*u_{k}(t)\right]e^{-j\omega_{k}t}\right\|}_{2}^{2}\right\},\;\\ s.t.\sum_{k}u_{k}(t)=x(t) \end{array}$$

where {*u_k_*} denotes *K* BLIMFs, and {*ω_k_*} denotes *K* center frequencies. The bandwidth of each mode is solved through the following steps:

(1) Hilbert transform is conducted on the decomposed *u_k_* to obtain the unilateral spectrum.
(2) The obtained unilateral spectrum is multiplied by the exponential function $e^{-j\omega_{k}t}$ to transform the frequency spectrum of each corresponding *u_k_* to the base frequency band.
(3) The gradient *L*^2^ is adopted to calculate the obtained *u_k_*.

To obtain the best solution of the restrictive problem in (1), the penalty factor *α* and Lagrange multiplication factor *λ*(*t*) are introduced to transform the variational restrictive problem into a non-restrictive problem. The substituted problem can be expressed as

(2) $$\begin{array}{l} L\left(\left\{u_{k}\right\},\left\{\omega_{k}\right\},\lambda \right)=\alpha \sum_{k}{\left\|\partial_{t}\left[\left(\delta (t)+\frac{j}{\mathrm{\pi t}}\right)*u_{k}(t)\right]e^{-j\omega_{k}t}\right\|}_{2}^{2}\\ \;\;\;\;\;+{\left\|x(t)-\sum_{k}u_{k}(t)\right\|}_{2}^{2}+\langle \lambda (t),x(t)-\sum_{k}u_{k}(t)\rangle \end{array}$$

To obtain the optimal solution of the non-restrictive model in Equation (1), the alternating direction multiplier algorithm (ADMM) is adopted to update the Lagrange multiplication factor *λ*(*t*), modes *u_k_*, and center frequency *ω_k_*. The specific algorithm is as follows:

(1) Initialization of $\left\{{\bar{u}}_{k}^{1}\right\}$, $\left\{{\bar{\omega }}_{k}^{1}\right\}$, and *λ*^1^.
(2) Minimization of *u_k_*, *ω_k_*, and *λ* by the following iteration, where *α* is the penalty factor.
  (3) $${\bar{u}}_{k}^{n+1}(\omega )=\frac{\bar{x}(\omega )-\sum_{i\neq k}{\bar{u}}_{i}(\omega )+(\bar{\lambda }(\omega )/2)}{1+2\alpha {\left(\omega -\omega_{k}\right)}^{2}}$$
  For a certain value of *K*, a smaller *α* leads to the mode mixing problem, and a larger *α* leads to a narrow passband, resulting in a larger error between the reconstructed signal and the original signal.
  (4) $$\omega_{k}^{n+1}=\frac{\int_{0}^{\infty }\omega {\left|{\bar{u}}_{k}(\omega )\right|}^{2}\mathrm{d\omega}}{\int_{0}^{\infty }{\left|{\bar{u}}_{k}(\omega )\right|}^{2}\mathrm{d\omega}}$$
  (5) $$\lambda^{n+1}=\lambda^{n}+\tau \left(x\cdot \sum_{k}u_{k}^{n+1}\right)$$
(3) The iteration steps are repeated to obtain *K* BLIMFs, and stop when the threshold range *e* is met: $\sum_{k}{\left\|{\bar{u}}_{k}^{n+1}-{\bar{u}}_{k}^{n}\right\|}_{2}^{2}/{\left\|{\bar{u}}_{k}^{n}\right\|}_{2}^{2}<e$, where *e* is the preset standard.

*ω_k_* is the center frequency in the BLIMFs, and ${\bar{u}}_{k}^{n+1}(\omega )$, $\bar{x}(\omega )$, $\bar{\lambda }(\omega )$ are Fourier transformation of its $u_{k}^{n+1}(\omega )$, *x*(*ω*), and *λ*(*ω*), respectively. VMD decomposes the signal into a set of BLIMFs denoting dynamic characteristics of the signal. From the perspective of signal separation, VMD formulates decomposition as a constrained variational optimization problem. Each mode is estimated simultaneously under a limited-bandwidth constraint, and its center frequency is updated during the iterative process. This mechanism differs from the recursive sifting procedure of EMD and the noise-assisted decomposition strategies of EEMD and CEEMDAN. For vibro-acoustic signals, the simultaneous optimization and bandwidth constraint of VMD can reduce the overlap between adjacent modes and improve the separation of modulation-related components. The Wiener-filter interpretation also provides a degree of noise suppression by restricting each mode to a specific frequency band. However, the performance of VMD depends on the selection of the mode number *K* and the penalty factor alpha. Therefore, the VMD results in this study should be interpreted as being obtained under the selected parameter configuration rather than as universally optimal decompositions.

### 2.2. Multidimensional Mean Gnome Entropy

Conventional entropies have an inevitable problem: they rely heavily on the selection of parameters, including dimension m and scale factor *r*. Studies show that small changes in parameters significantly affect the entropy calculation results. Recently, Manis et al. [51] proposed “Bubble entropy” to eliminate the determination of *r*, which is a major step forward in entropy theory. Wang et al. [52] then proposed GE, which uses Gnome sort instead of Bubble sort algorithm in ascending order. The steps of GE are as follows:

(1) A time series *x* = *x*_1_,*x*_2_,…,*x_N_* is reconstructed to *m*-dimensional phase space by phase space reconstruction. The reconstructed time series is
  *X* = *X*_1_,*X*_2_,...,*X_N_*_−*m*+1_ (6)
  where *X_i_* = (*x_i_*,*x_i_*_+1_,…,*x_i_*_+*m*−1_).
(2) Each vector *X_i_* is sorted by the Gnome sort algorithm, which is also called stupid sort, in ascending order, and the necessary swaps *n_i_* are counted. The Gnome sorting algorithm is an adjacent-swap sorting algorithm that starts from the first pair of adjacent elements. If the current pair is in ascending order, the pointer moves forward by one position. If the pair is not in ascending order, the two elements are exchanged, and the pointer moves backward by one position. When the pointer is at the first position, it remains there after an exchange. The algorithm is completed when the pointer reaches the last position of the vector. The maximum swap number is computed as *m* × (*m* − 1)/2. Then, the probabilities of *n_i_* can be expressed as
  (7) $$p_{i}=\frac{n_{i}}{m\times (m-1)/2}$$
(3) The Renyi permutation entropy [53] is computed as
  (8) $${RpE}^{m}=\frac{1}{1-\alpha }\log\left(\sum_{i=1}^{n}p_{i}^{\alpha }\right)$$
  where *α* = 2 obtains the best performance based on prior investigation.
(4) The GE can be computed as follows:
  (9) $$GE^{m}=\frac{{RpE}^{m+1}-{RpE}^{m}}{\log\left[\left(m+1\right)/(\left.m-1\right)\right]}$$
(5) Similarly, the value of *GE^m^*^+1^ is computed by repeating the steps and updating *m* to *m* + 1. Thus, multidimensional Gnome entropy values are obtained.

The effectiveness and superiority of GE has been verified in previous studies [52], although there are still some shortcomings. For example, the entropy could be negative under some circumstances, and it lacks stability with the increase in multiple dimensions. Here, MGE is proposed to improve GE. MGE solves the issue of existing negative values by bringing the Heaviside function to eliminate the low amplitude data. Thus, the mean processing in MGE improves the accuracy and stability of GE values in multiple dimensions. The updated computation steps of MGE are shown as follows:

(1) For a signal *x* = *x*_1_,*x*_2_,…,*x_N_*, the Heaviside function is adopted to enhance the characteristic information of signals and remove negative entropy values. The processing is carried out as follows:
  (10) $$\bar{x_{i}}=x_{i}H(x_{i})$$
  where
  (11) $$H(x_{i})=\left\{\begin{array}{cc} 0 & x_{i}<\mathrm{std}(x)\\ 1 & x_{i}>\mathrm{std}(x) \end{array}\right.$$
(2) The time series *x* is reconstructed to *m*-dimensional phase space. The reconstructed time series is
  *X* = *X*_1_,*X*_2_,...,*X_N_*_−*m*+1_ (12)
  where *X_i_* = (*x_i_*,*x_i_*_+1_,…,*x_i_*_+*m*−1_).
(3) Each vector *X_i_* is sorted by the Gnome sort algorithm in ascending order. The number of necessary swaps is counted as *n_i_*. The maximum swap number is *m* × (*m* − 1)/2. The probabilities of *n_i_* can be expressed as
  (13) $$p_{i}=\frac{n_{i}}{m\times \left(m-1\right)/2}$$
(4) The value of *p_i_* is taken as permutation probabilities, and the MGE values can be computed as follows:
  (14) $${MGE}^{m}=\frac{1}{N-m+1}\sum_{i=1}^{n}p_{i}$$

Multidimensional MGE values can be obtained to characterize the dynamical characteristics of the signal. The proposed MGE solves the issue of negative values in the GE algorithm. It improves the accuracy and stability of GE values in entropy calculation by mean processing on multiple dimensions. MGE reflects the complexity and regularity of the vibro-acoustic signals, an can be used in the quantitative measurement of vibro-acoustic signals.

VMD formulates signal decomposition as a constrained variational optimization problem. Unlike the recursive sifting procedure of EMD and the noise-assisted strategies of EEMD and CEEMDAN, VMD estimates multiple modes simultaneously under a bandwidth constraint. This property helps reduce the overlap between adjacent modes and improves the separation of different frequency components in vibro-acoustic signals. In addition, the Wiener-filter interpretation of VMD can reduce the influence of broadband noise when suitable parameters are selected. However, the decomposition performance is affected by the mode number *K* and the penalty factor *α*. Therefore, the results of this study represent the performance of VMD under the selected parameter configuration and should not be regarded as universally optimal.

### 2.3. Convolutional Neural Network

CNN is one of the leading deep learning techniques used in feature extraction. The typical structure of a CNN model consists of three main layers: the convolutional layer, pooling layer, and fully connected layer [54]. The convolutional layer detects the local conjunctions of features from the previous input layer, and involves a lot of feature learning. The pooling layer merges similar features together, thus reducing the data dimensionality. By processing the convolutional layer and pooling layer, the deep feature maps can be obtained. The fully connected layer is used to connect all neurons in the previous layers to acquire global semantic information. The lower level features in the CNN represent higher level features and are used to conduct deep learning.

**Figure 1:**
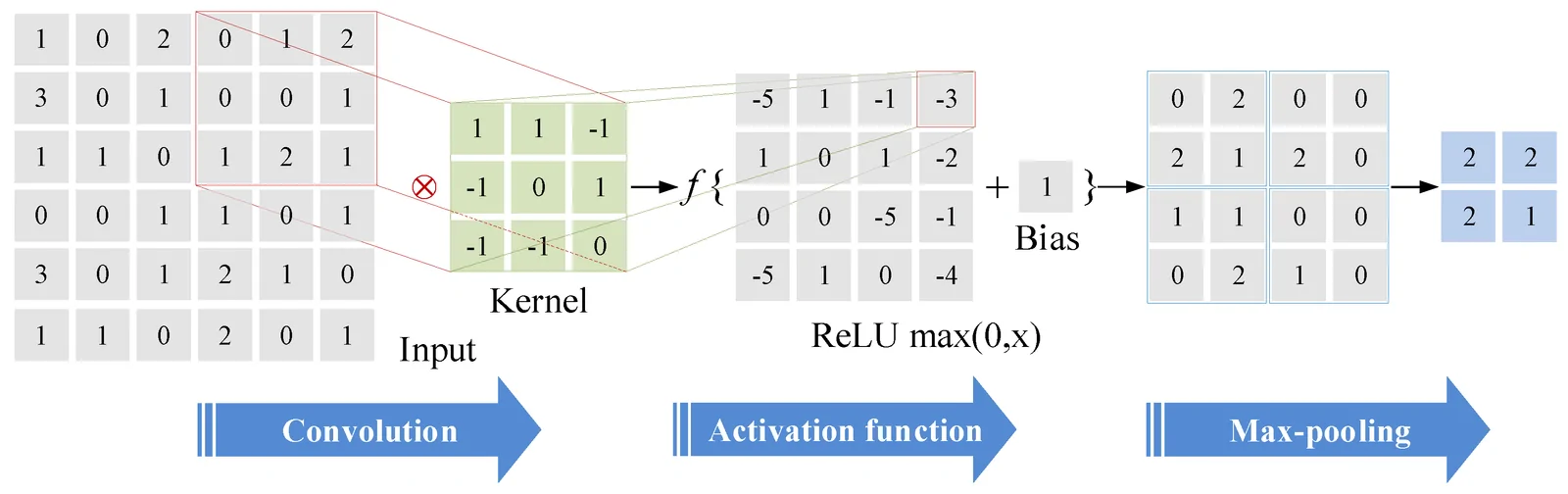
An example for illustration of CNN methodology.

During image processing, the motifs assembled into parts are formed by local combinations. The CNN performs high-level reasoning to conduct the identification. The local combinations of components are hierarchically composed across multiple layers to enable pattern recognition. During signal processing, a similar strategy is employed catering to the prerequisites of CNN. It has been successfully used in voice recognition [55] and mechanical fault diagnosis [56,57]. In this study, the feature matrices extracted from the one-dimensional vibro-acoustic signal are inputted into the CNN model. The simplified methodology of the typical CNN model is illustrated in Figure 1.

## 3. The Proposed Vibro-Acoustic Fault Diagnosis Approach via IMGE Analysis and CNN

### 3.1. Intrinsic Multidimensional Gnome Entropy Analysis

When different defects occur in the important parts of rotary machinery, such as gears and bearings, the vibro-acoustic signals have different dynamic characteristics. Studies of EMD- and VMD-derived algorithms show that IMFs and BLIMFs represent signal dynamics and the underlying properties of decoupled modulation frequency information [24,25,58,59]. VMD decomposes the signal into a set of BLIMFs with limited frequency bands, which have the specific sparsity properties of its bandwidth in the spectral domain, and the filter bank property. The proposed MGE solves the issue of negative values in GE by using the Heaviside function to eliminate low-amplitude data. MGE improves the accuracy and stability of GE in multiple dimensions and reflects the signal’s complexity and regularity. The authors’ previous studies [59] indicate that intrinsic characteristics of the signal can be extracted from the modes by measuring its complexity and regularity.

This paper proposes the strategy of IMGE analysis to extract quantitative features of the vibro-acoustic signals. IMGE analysis consists of signal decomposition by VMD and feature extraction by the proposed MGE. VMD is used to decompose the vibro-acoustic signals into BLIMFs that denote dynamical characteristics. The multidimensional entropy values of BLIMFs are adopted as the quantitative features.

### 3.2. The Proposed Vibro-Acoustic Fault Diagnosis Approach

This paper proposes a novel vibro-acoustic approach to fault diagnosis using IMGE analysis and CNN. The vibro-acoustic signals are collected by microphones or smartphones during daily use. The main steps of the proposed vibro-acoustic approach are as follows:

(1) A microphone or smartphone is used to collect the vibro-acoustic signals of different types of bearings and gears. The vibro-acoustic signals describe characteristic fault information, just like the vibration signals collected by acceleration sensors. Vibro-acoustic signal processing is a non-destructive technology.
(2) The vibro-acoustic signals are decomposed into BLIMFs, characterizing the dynamics of the signals. n-dimensional MGE values of n-order BLIMFs are computed as quantitative features. Thus, the feature matrix is extracted to denote the vibro-acoustic signals.
(3) For each vibro-acoustic signal sample, an *n* × *n* feature matrix is obtained. Feature matrices are used as training and testing samples in the CNN. Deep feature maps of the inputs are obtained, and the output layer denotes different patterns of rolling bearings and gears. CNN achieves good and stable identification results, which contributes to the accurate diagnosis of faults in rotary machinery.

**Figure 2:**
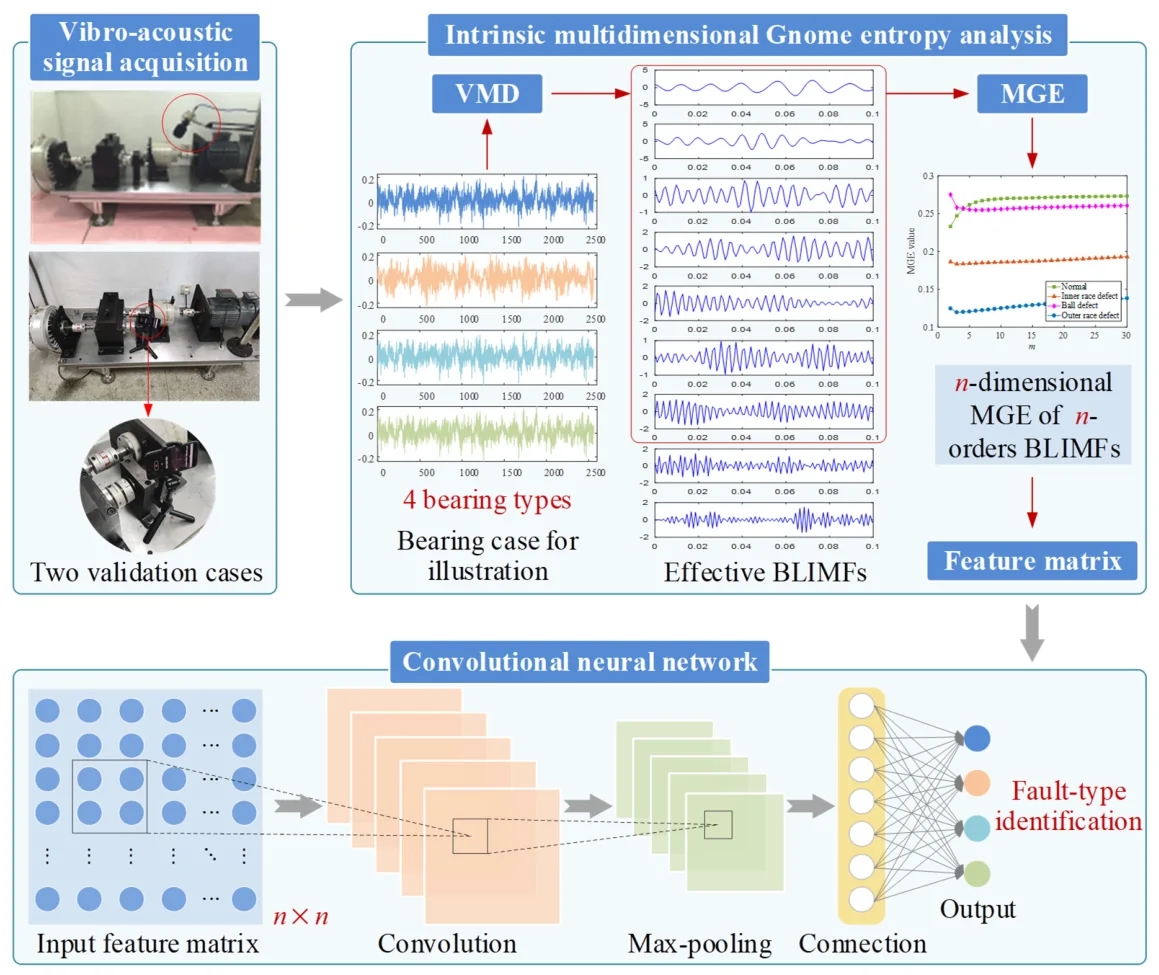
The proposed vibro-acoustic fault diagnosis approach via IMGE analysis and CNN.

A graphical illustration of the proposed vibro-acoustic fault diagnosis approach is shown in Figure 2. Each vibro-acoustic signal is first divided into segments containing 4096 sampling points. Each segment is decomposed by VMD into a set of BLIMFs. The first seven effective BLIMFs are retained, and the MGE values corresponding to embedding dimensions 11–17 are calculated for each selected BLIMF. Consequently, each signal segment is represented by a 7 × 7 IMGE feature matrix. The feature matrix is then used as the input of the CNN, and the Softmax output provides the predicted condition label. Therefore, VMD performs mode separation, MGE constructs the quantitative feature representation, and CNN performs fault-pattern classification.

## 4. Numerical Simulation and Effect Validation

### 4.1. Numerical Simulation of Variational Mode Decomposition

**Figure 3:**
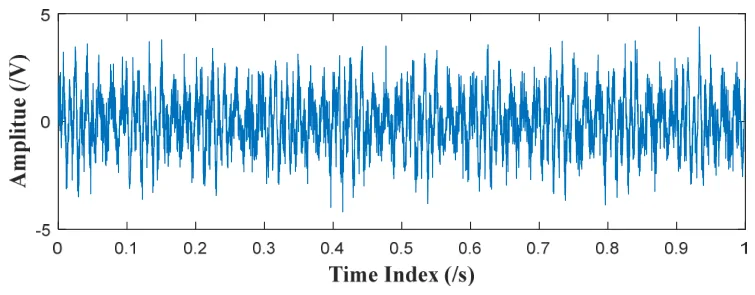
Time domain plot of the simulated signal.

Under practical conditions, fault characteristic frequencies are altered by the structural and modulation frequencies. Amplitude- and frequency-modulated signals are used here to construct the simulated signal of a faulty rolling bearing. The simulated signal is simplified to the signal model as follows:

*x*(*t*) = *α*sin(2*πf_b_t*)[1 + *β*cos(2*πf_r_t*)] (15)

where *f_b_* and *f_r_* denote the fault characteristic frequency and rotational frequency, respectively. *α* and *β* denote the signal’s power amplitude. To simulate additive noise, Gaussian white noise *n*_1_ with a variance of 0.5 and average of 0 was added. The simulated signal can be expressed as follows:

*x*(*t*) = sin(2*πf*_1_*t*) + cos(2*πf*_2_*t*)[1 + 0.8sin(2*πf*_3_*t*)] + *n*_1_ (16)

where *f*_1_, *f*_2_, and *f*_3_ are 55, 120, and 10 Hz, respectively. For numerical simulation, the sampling frequency and sampling points are 4096 Hz and 4096, respectively. The time domain plot of the simulated rolling bearing signal *x*(*t*) is presented in Figure 3.

**Figure 4:**
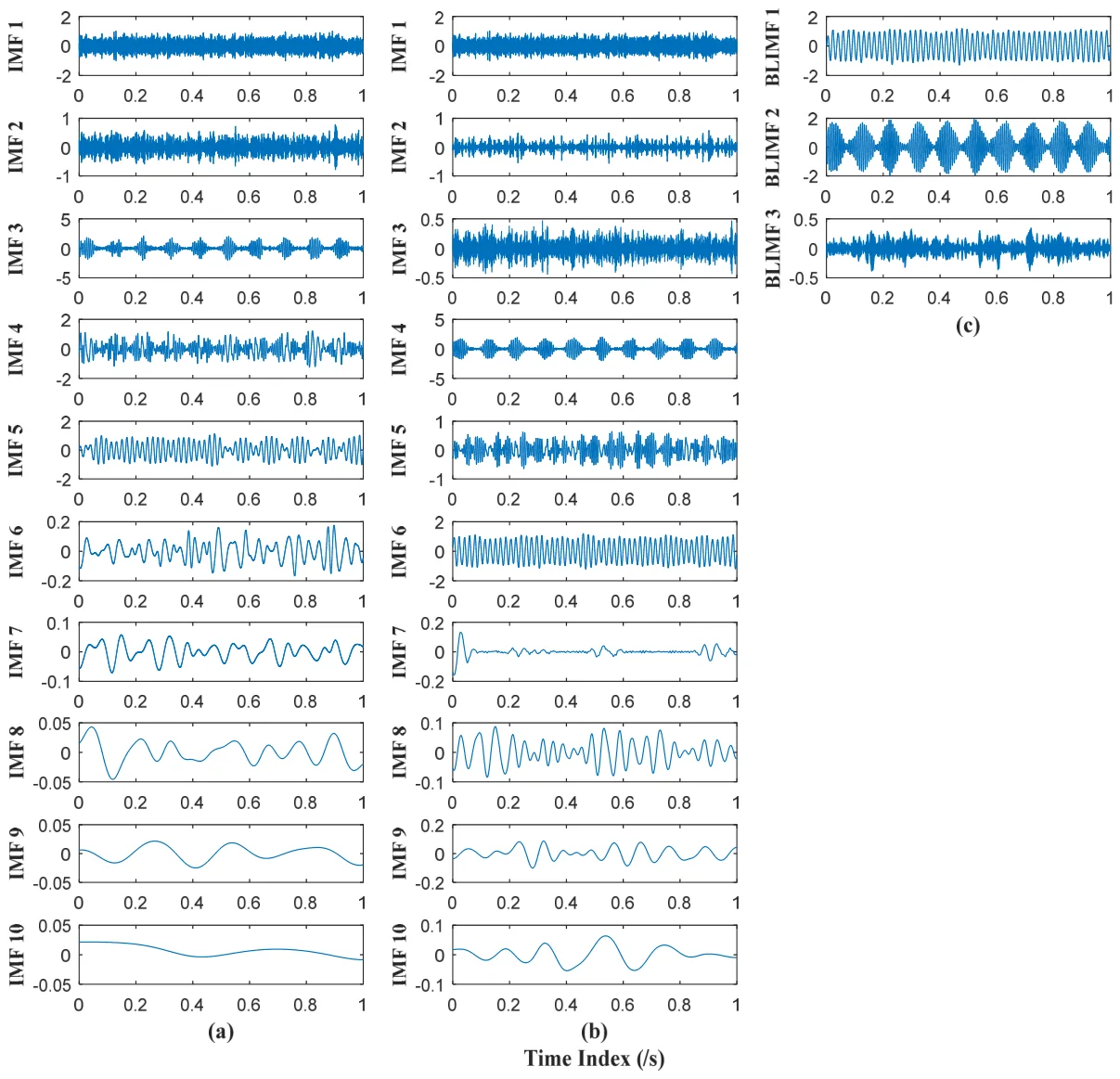
Time domain plots of the obtained modes: (**a**) EEMD, (**b**) CEEMDAN, and (**c**) VMD.

**Figure 5:**
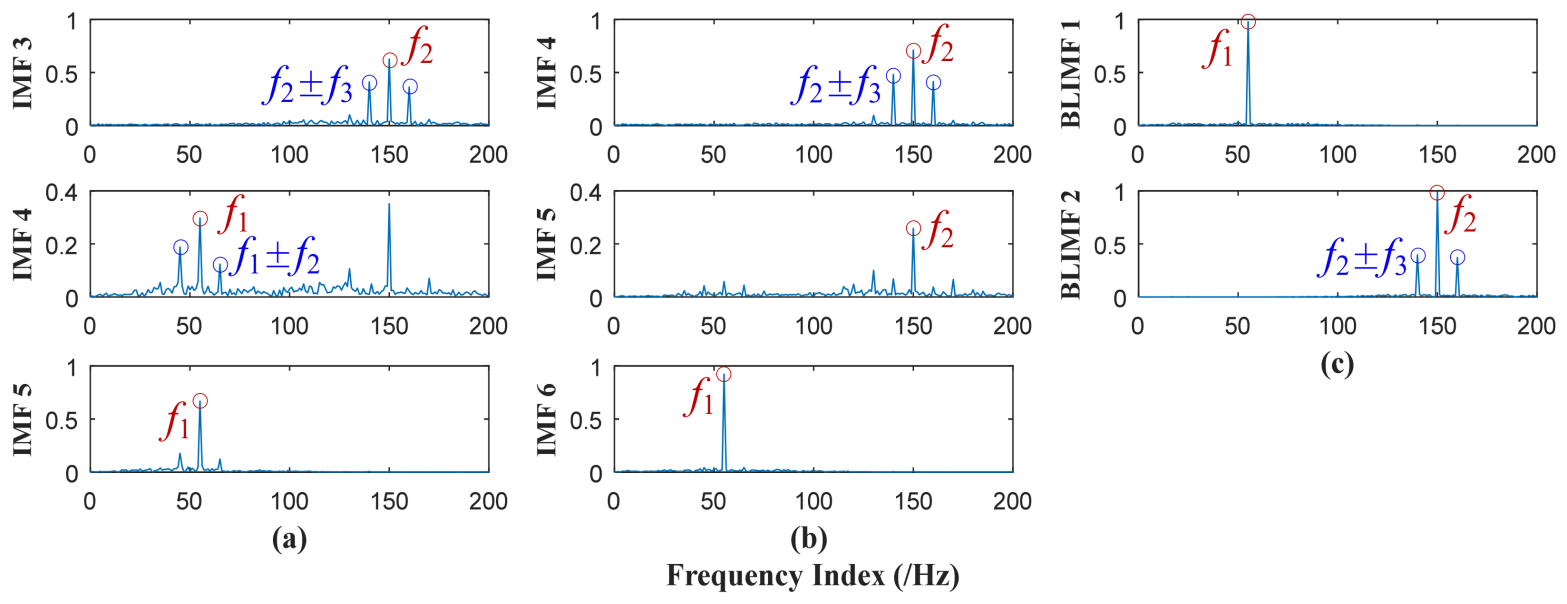
Frequency domain plots of effective modes: (**a**) EEMD, (**b**) CEEMDAN, and (**c**) VMD.

EEMD and CEEMDAN were employed to process the signal. Before decomposition, each signal segment was normalized by its standard deviation. For EEMD, the amplitude of the added white noise was set to 0.2 times the standard deviation of the normalized signal, and the ensemble size was set to 50. For CEEMDAN, 25 positive–negative noise pairs were used, corresponding to 50 noise-assisted realizations. The same noise-amplitude scale was used for EEMD and CEEMDAN to ensure a consistent comparison. The theories of these algorithms have been illustrated in the authors’ previous studies [59]. In VMD, a smaller *K* causes mode mixing, and a larger *K* generates redundant modes. For a certain *K*, a smaller *α* causes mode mixing, while a lager *α* leads to narrow passband, resulting in larger reconstruction error. Here, these parameters were determined by the Grasshopper optimization algorithm [35], and *K* and *α* were determined as 3 and 5000. The time domain plots of IMFs by EEMD and CEEMDAN, and BLIMFs by VMD, are shown in Figure 4a–c. The effective modes are determined by the fault correlation factor analysis [57]. The frequency domain plots are shown in Figure 5a–c.

Figure 4a,b presents the time domain plots of the first 10 IMFs obtained by EEMD and CEEMDAN, respectively, while Figure 4c shows the three BLIMFs obtained by VMD. In the IMFs obtained by EEMD and CEEMDAN, there are still redundant modes not denoting characteristic frequencies. The frequency domain plots of effective modes are shown in Figure 5. In the 4th IMF obtained by EEMD, mode mixing is present, along with the wrong characteristic frequencies *f*_1_ ± *f*_2_. *f*_2_ exists in the 4th and 5th IMFs obtained by the CEEMDAN, while for VMD, *f*_1_ appears in the 1st BLIMF, and *f*_2_ and *f*_2_ ± *f*_3_ appear in the 2nd BLIMF without interference frequencies. It can be concluded from the above analysis that VMD is superior to traditional algorithms and thus can be used to diagnose faults in rotary machinery. To ensure a fair comparison, the decomposition parameters were determined before classification and were kept fixed for all methods evaluated on the same dataset. EEMD and CEEMDAN used the same noise-amplitude scale, while the ensemble realizations were fixed throughout the corresponding experiments.

### 4.2. Effect Validation of Multidimensional Mean Gnome Entropy by Case Western University Bearing Dataset

**Figure 6:**
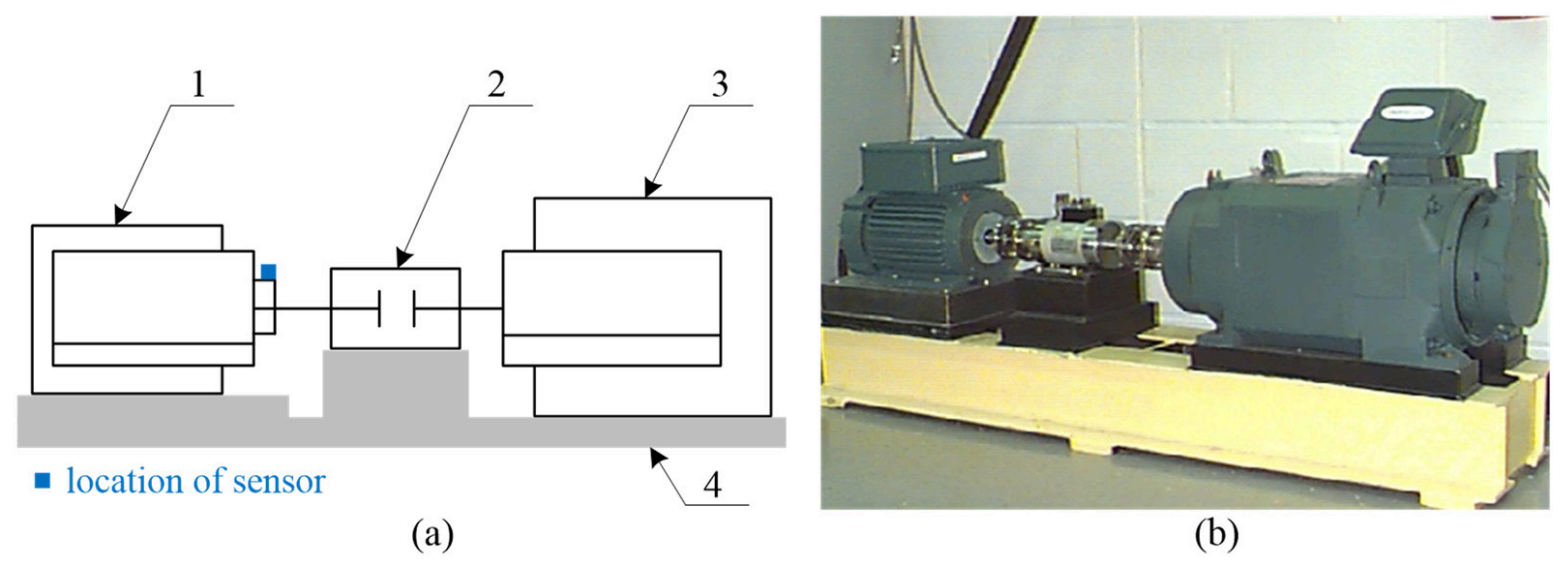
(**a**) 1—motor, 2—torque transducer/encoder, 3—dynamometer, and 4—base plate; (**b**) the apparatus.

The effectiveness of GE has been validated in previous research [52]. The faulty bearing datasets from Case Western Reserve University Bearing Database [60] were adopted to verify the effectiveness or improved performance of the proposed MGE. An electro discharge machine was used to generate different faulty bearings. The sampling frequency was 12 kHz. The apparatus and its photo are shown in Figure 6.

**Figure 7:**
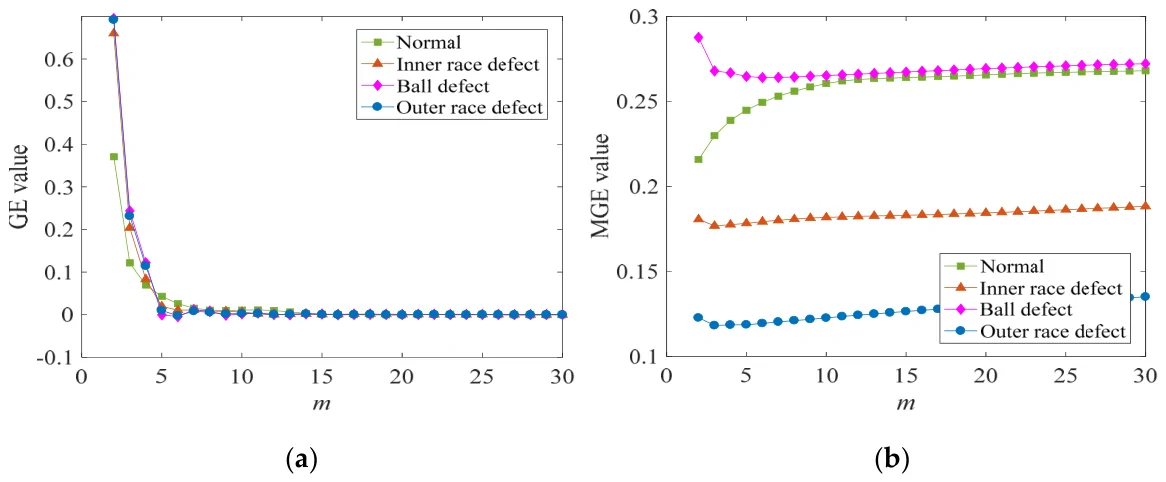
(**a**) GE and (**b**) MGE on multiple dimensions, of vibration signals with the length of 4096 points.

**Figure 8:**
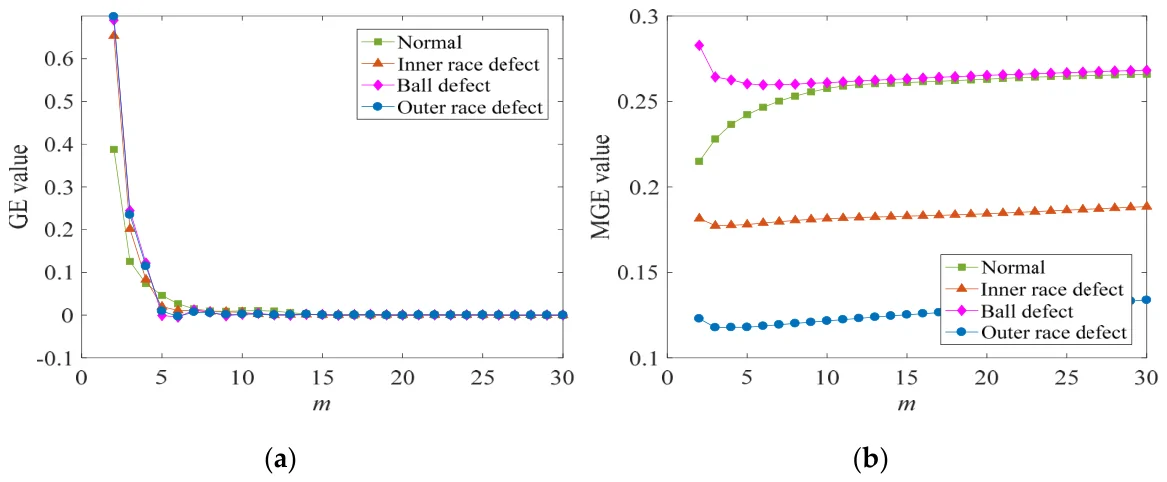
(**a**) GE and (**b**) MGE, on multiple dimensions, of vibration signals with the length of 8192 points.

Data samples of faulty bearings were used. The defect diameter was 0.007 inch, and the motor load and motor speed were 2 Hp and 1750 rpm. The dimension m was chosen as 30 to reflect the complexity and regularity of the vibration signals. The GE and MGE values of vibration signals, with different lengths, are shown in Figure 7 and Figure 8.

**Figure 9:**
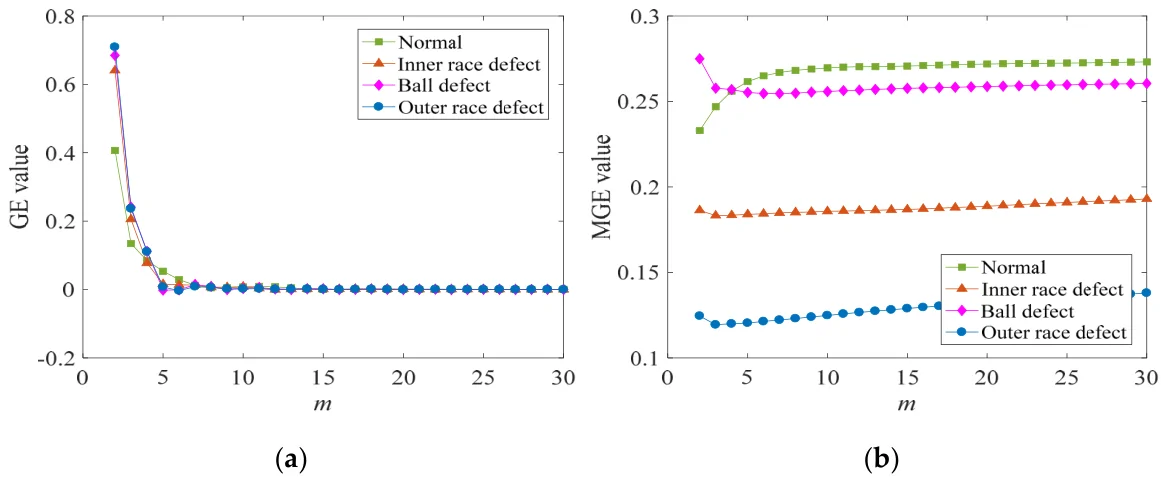
(**a**) GE, (**b**) MGE, on multiple dimensions, of vibration signals with the length of 4096 points.

Figure 7 and Figure 8 illustrate that GE values decrease monotonically across four bearing health conditions: normal state, inner race fault, ball fault, and outer race fault. As the scale increases, the entropy values obtained by GE for these four rolling-bearing cases overlap heavily, which hinders the identification of bearing fault types. By contrast, MGE yields clearly distinguishable values between the four bearing conditions. Such behavior holds for signal segments of 4096 points and 8192 points. Repeated tests reveal that the performance of MGE is not sensitive to signal segment length. Balancing complexity measurement capability and computational cost, we selected 4096 as the signal sample length for subsequent processing. To further verify the validity of MGE, bearing signals under different working conditions were analyzed, with a motor load of 3 Hp and a rotational speed of 1730 rpm. The corresponding GE and MGE results are plotted in Figure 9.

**Figure 10:**
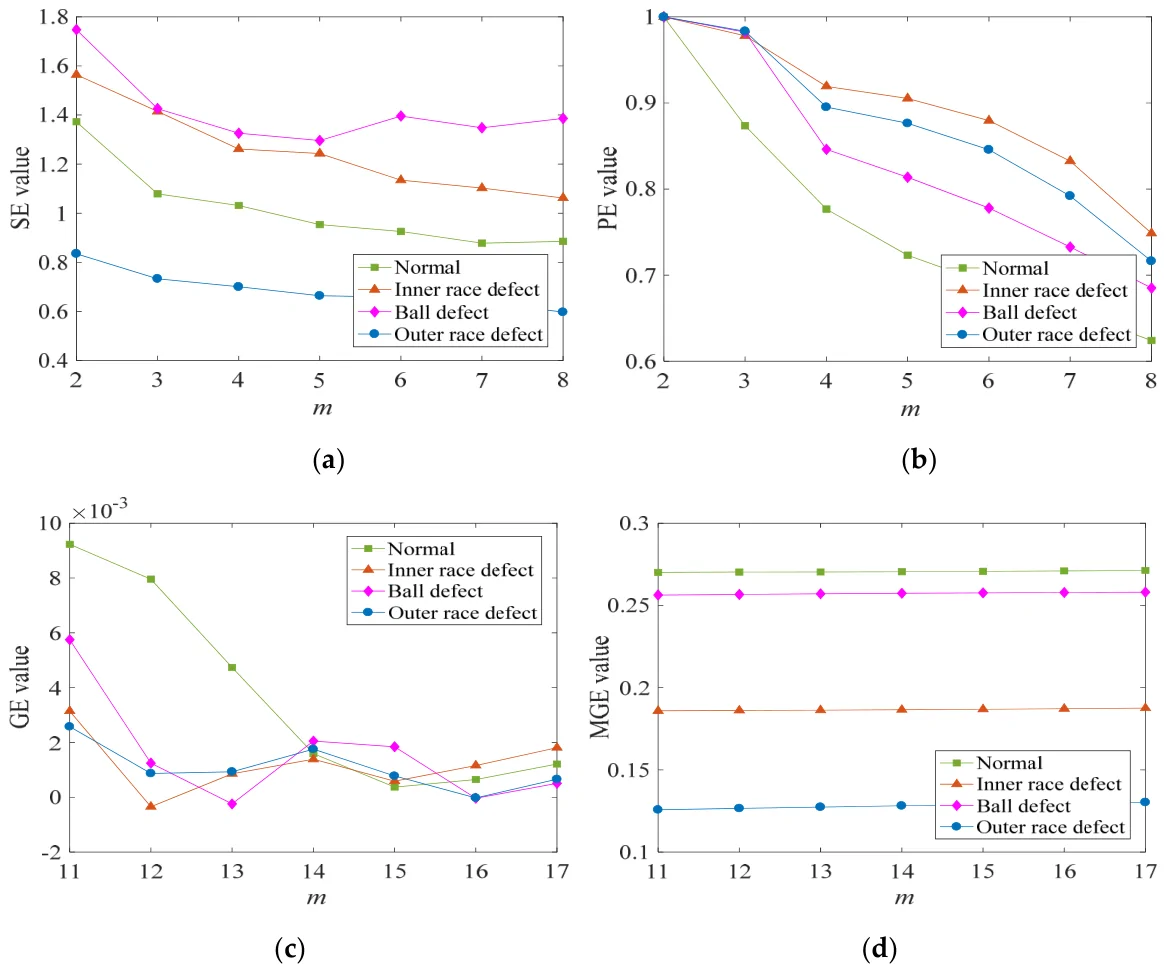
(**a**) SE, (**b**) PE, (**c**) GE, and (**d**) MGE, on multiple dimensions, of vibration signals with the length of 4096 points.

Figure 9 shows that multidimensional MGE values denoting four types of bearings show significant difference, while GE values cannot achieve the intended effect. The analysis between GE and MGE under various operating conditions illustrates the effectiveness or improved performance of MGE. SE and PE of the data samples with the length of 4096 points are analyzed to illustrate the validity of MGE. Four kinds of entropy values of signals with 4096 points are shown in Figure 10.

The results show that MGE produces more distinguishable feature values than GE under the tested conditions. This result demonstrates improved empirical discriminability, but should not be interpreted as a universal proof that MGE is a more accurate complexity measure. The distinction between four types bearings is significant. Figure 7, Figure 8 and Figure 9 show that multidimensional MGE tends to have a good classification effect starting with the dimension of 6. In this paper, 7 BLIMFs will be adopted, and so MGE values on 7 dimensions were used to construct a 7 × 7 feature matrix. Entropy values with the dimension of 11–17 are analyzed. Figure 10 shows that between four types of rolling bearings, SE and PE values have an effect on fault identification under the same operating conditions. GE values show some turbulence with the increase in dimension, and multidimensional MGE values show a distinctive difference between the four types of rolling bearings. In addition, SE, PE, and GE values change with the increase in dimension, while MGE values show good stability with the increase in dimension. The classification effect and stability property of the proposed MGE algorithm contribute to fault diagnosis of rotary machinery.

## 5. Application Studies

Two experimental cases were used to verify the proposed vibro-acoustic fault diagnosis approach. The gear and bearing datasets collected from the diagnosis simulator were analyzed. A microphone or smartphone was used to collect vibro-acoustic signals from machinery with normal and defective parts (the details of faulty gear and rolling bearings and vibro-acoustic signal acquisition equipment are provided later). For both cases, the random data partition was used to evaluate within-dataset classification performance under the investigated laboratory conditions, rather than independent-session generalization.

### 5.1. Case 1: Application to Gear Dataset Processing of Drivetrain Diagnosis Simulator

The vibro-acoustic signals of faulty gears collected from the planetary gearbox diagnosis simulator were analyzed. The apparatus was produced by Zhenjiang Tianyu Technology Inc, and the external bearing pedestal was customized as requested by the authors on a test bench. The apparatus consists of a variable speed motor, a helical gearbox, a planetary gearbox, an external bearing pedestal, and a magnetic powder brake. The large and small gears of the helical gearbox have 77 and 55 teeth, respectively. The planetary gear ring, planetary gear, and sun gear have 72, 27, and 18 teeth, respectively, and there are 3 planetary gears. The bearing pedestal is equipped with a cylindrical rolling bearing of the model HRB-N205M. The apparatus can simulate gear faults and rolling bearing faults. This experiment was conducted to verify the effectiveness or improved performance of the proposed vibro-acoustic approach to fault diagnosis in gears.

**Figure 11:**
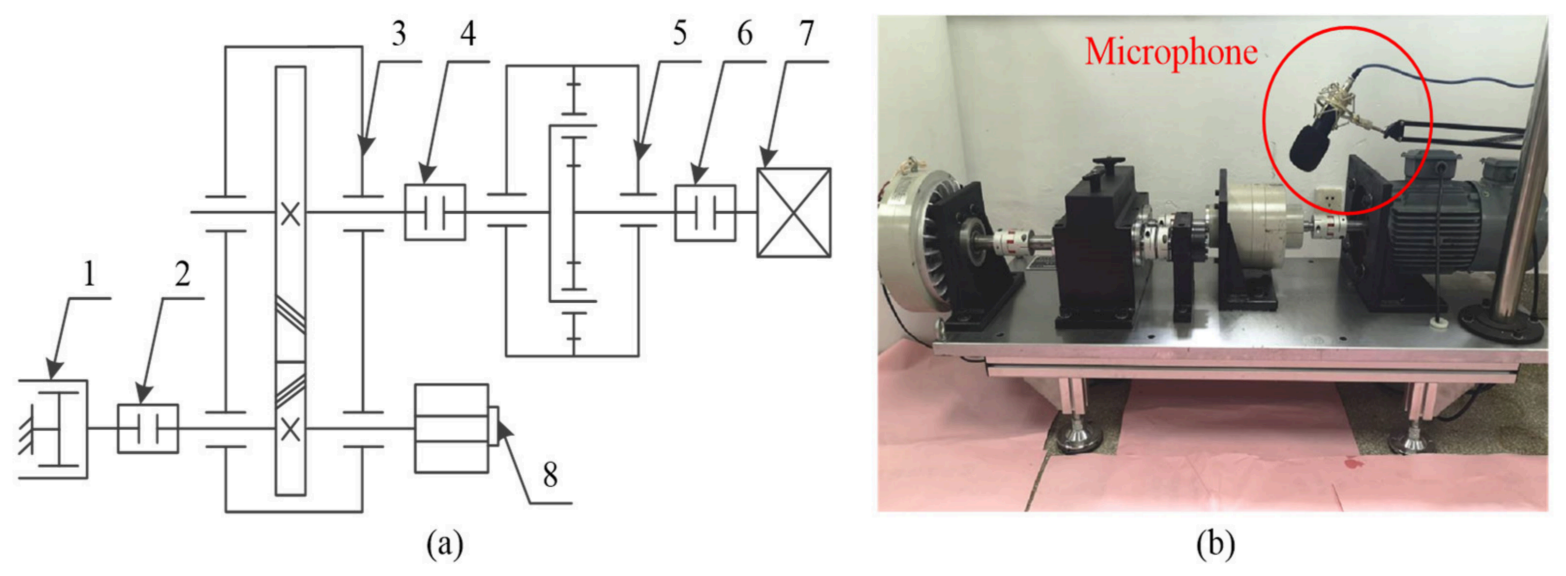
(**a**) The schematic diagram of the experimental apparatus, with 1—magnetic powder brake, 2, 4, 6—shaft coupling, 3—helical gear box, 5—planetary gear box, 7—variable motor, and 8—external bearing pedestal. (**b**) A picture of the experimental apparatus and signal acquisition equipment.

The microphone was used to collect vibro-acoustic signals from four kinds of gears, including normal gears and faulty gears with wear, broken teeth, and cracked teeth. The motor speed was 1500 r/min, and the sampling frequency was 8192 Hz. One-hundred samples of each defect with the length of 4096 points were acquired. The schematic diagram and the experimental apparatus along with the data acquisition equipment are shown in Figure 11.

VMD was used to decompose the vibro-acoustic signals. At this point, the mode order and penalty factor were chosen. During the decomposition, a smaller *K* causes the mode mixing problem, and a larger *K* generates redundant modes. For a certain value of *K*, a smaller *α* would cause the mode mixing problem, and a larger *α* leads to a narrow passband, resulting in a larger error between the reconstructed signal and the original signal. In this study, the parameters were determined by Grasshopper optimization algorithm [35]. *K* and *α* were determined as 10 and 5000.

**Figure 12:**
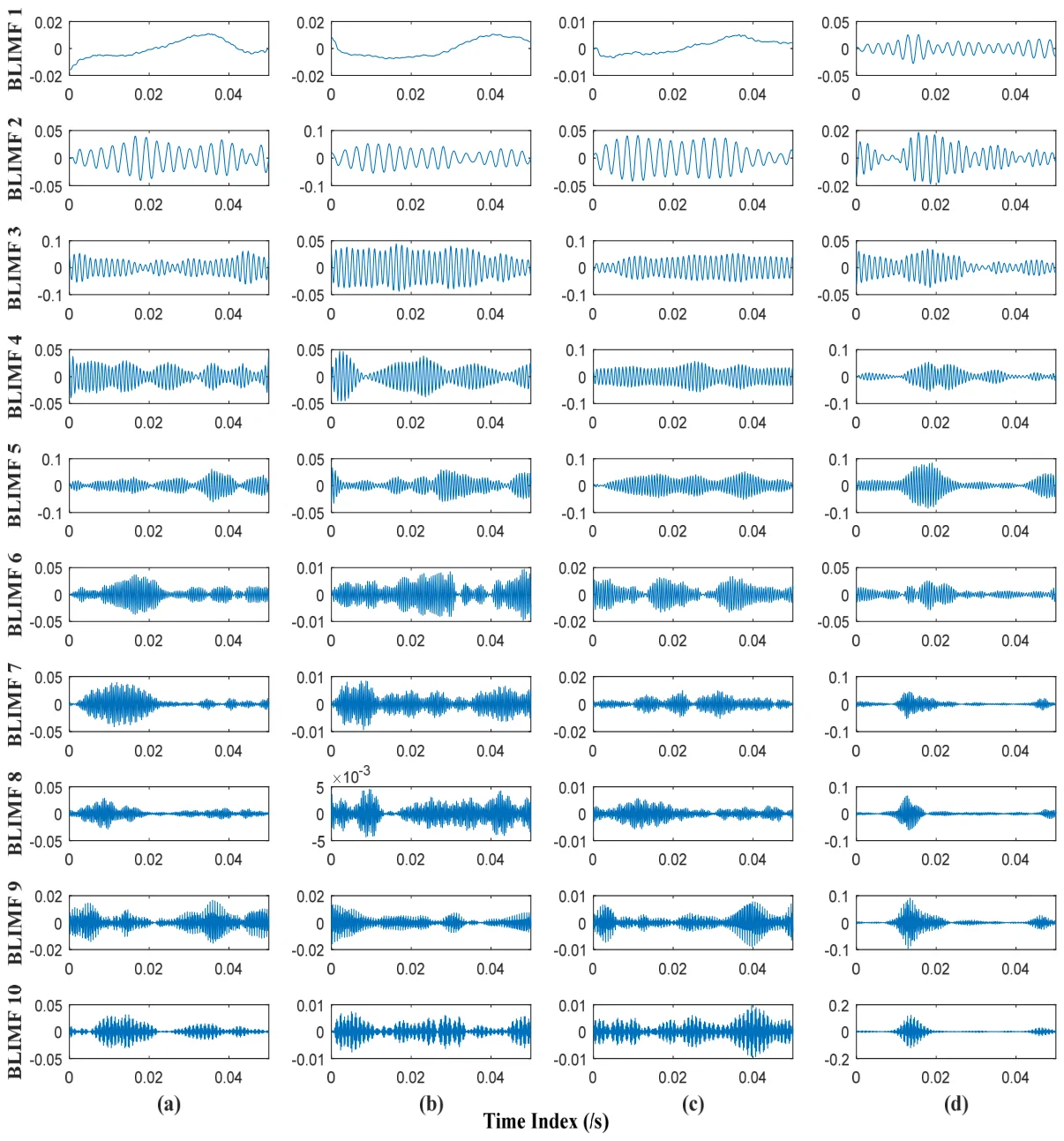
Time domain plots of the first 10 BLIMFs obtained by VMD from the vibro-acoustic signals of different gear conditions: (**a**) normal gear, (**b**) gear wear, (**c**) cracked tooth, and (**d**) broken tooth.

**Figure 13:**
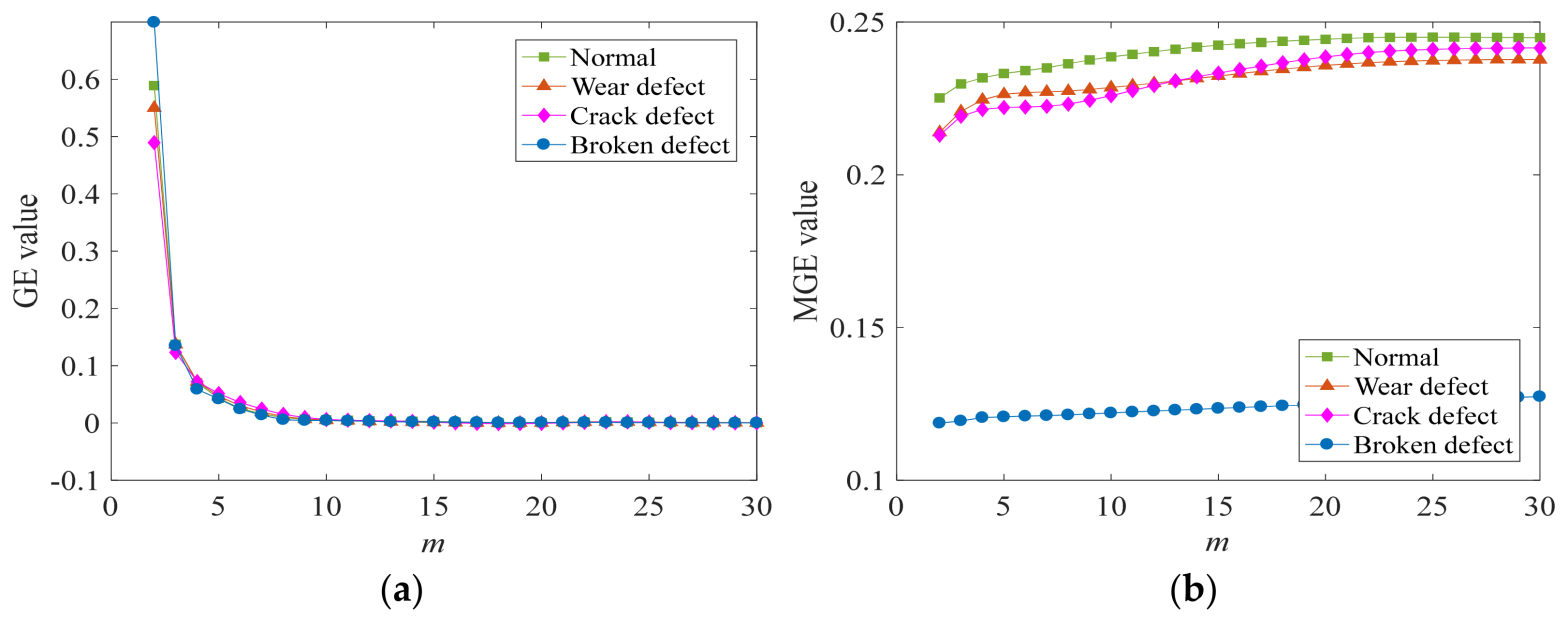
(**a**) GE values, (**b**) MGE values, on multiple dimensions, of 7th BLIMF with the length of 4096 points.

Figure 12 shows the 10 BLIMFs obtained from the vibro-acoustic signals of the four gear conditions. MGE was used to measure the complexity and regularity of the BLIMFs. The dimension *m* was set at 30 to reflect the complexity and regularity of the vibro-acoustic signals. To diagnose faults in gears, the MGE values of BLIMFs were adopted as quantitative features. The GE and MGE values of the seventh BLIMF were computed and are shown in Figure 13.

Figure 13 shows that the GE values decreased monotonically for four types of gears, including normal gears, and faulty gears with wear, cracks, and broken teeth defects. The entropy values of the seventh BLIMF of four kinds of gears are mixed. The MGE values of the seventh BLIMF denoting different types of gears are very different. It can be concluded that BLIMFs characterize the dynamic property of the vibro-acoustic signals. The analysis illustrates the effectiveness and improved performance of MGE in quantifying the complexity and regularity of the vibro-acoustic signals.

**Figure 14:**
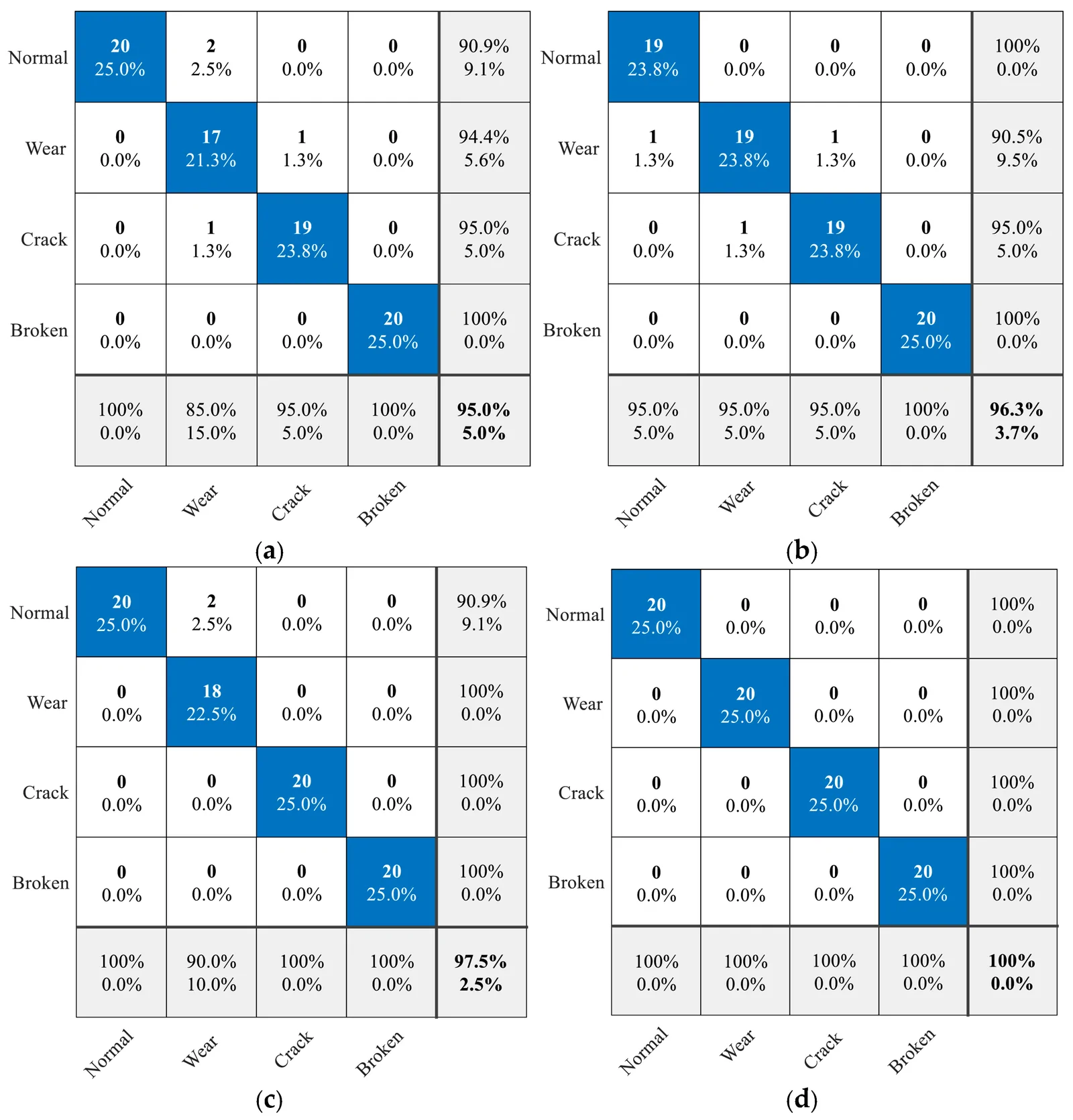
Confusion matrices of testing results by different strategies: (**a**) EEMD and GE, (**b**) CEEMDAN and GE, (**c**) IMGE analysis using VMD and GE, (**d**) IMGE analysis using VMD and MGE.

Then, 11–17 dimensional MGE values of the first seven orders BLIMFs were computed as quantitative features. The 7 × 7 feature matrices were extracted from all 100 vibro-acoustic signal samples, and then inputted into the CNN. The output of the CNN model is a vector having four elements, representing four types of gears. Deep learning is conducted by the convolutional layer, which has a 356-element two-dimensional filter, a max-pooling layer, and a two-element subsampling mask. The fully connected layer has 512 units, and is used to classify the learned features. The Softmax activation function is used in the output layer, and the ReLU function is employed in the other layers. The CNN was trained for 1000 epochs with minibatch gradient descent. For the training process, the categorical cross-entropy error and Adam optimizer was used, and the learning rate was 0.0001. For each gear condition, 100 signal segments with a length of 4096 points were obtained. These segments were randomly divided into a training set, a validation set, and a test set, containing 60, 20, and 20 segments, respectively. This protocol was used to evaluate the within-dataset classification performance under the fixed laboratory configuration and operating conditions. Because the available dataset was collected under a single controlled experimental setup, independent-session, cross-device, and cross-condition evaluations were beyond the scope of the present work. Therefore, the reported results should be interpreted as benchmark performance rather than as a direct evaluation of generalization to unseen operating conditions. The confusion matrices of the different test results are shown in Figure 14.

**Figure 15:**
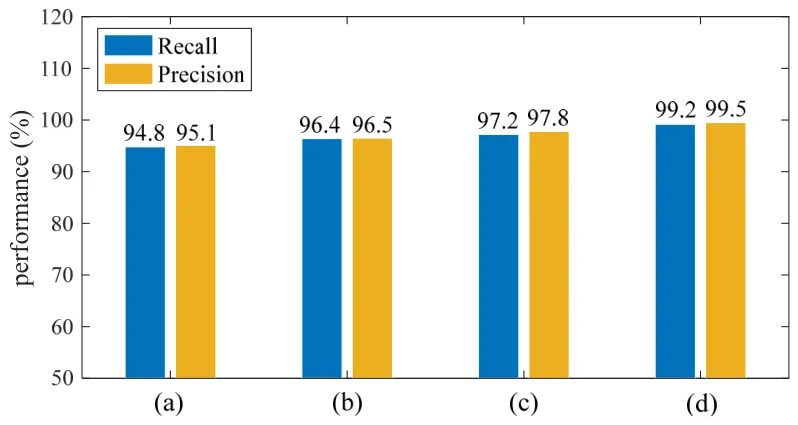
The average classification performance by different strategies: (**a**) EEMD and GE, (**b**) CEEMDAN and GE, (**c**) IMGE analysis using VMD and GE, (**d**) IMGE analysis using VMD and MGE.

Figure 14a shows that the testing accuracy of CNN by using EEMD and GE is 95.0%. The gear dataset shows proper effect in fault identification. Figure 14b shows that the testing accuracy achieved by using CEEMDAN and GE is 96.3%. Figure 14c shows that the accuracy achieved by IMGE analysis using VMD and GE is 97.5%. Figure 14d shows that the testing accuracy of the CNN using the proposed IMGE analysis with VMD and MGE reaches 100% under the experimental conditions and data partition adopted in this study. This result indicates that the extracted MGE features provide clear discriminative information for the four gear conditions considered here. However, the 100% testing accuracy should be interpreted within the specific laboratory conditions, signal acquisition settings, and dataset used in this study, rather than as a universally achievable accuracy for different operating conditions or unseen datasets. To evaluate the performance of the proposed approach accurately, the classification processes were both conducted 20 times. The mean values of recall and precision ratios are given in Figure 15.

Figure 15 shows that recall values obtained using EEMD and GE, along with CEEMDAN and GE, are 94.8% and 96.4%, respectively, while their precision values are 95.1% and 96.5%, respectively. This comparison verifies the logic of the proposed method. The quantitative features of the sub-signals depict dynamic characteristics of the signals. Thus, they can be used as the input for the neural network. The comparison between the strategies of (a), (b), and (c) validates the effectiveness or improved performance of VMD in processing the vibro-acoustic signals of faulty gears. The recall and precision values of adopting IMGE analysis using VMD and MGE are 99.2% and 99.5%. These results demonstrate that the proposed method provides the best classification performance among the compared strategies under the experimental conditions considered in this study. The studies show that the proposed vibro-acoustic approach to fault diagnosis in gears is promising.

### 5.2. Case 2: Application to Bearing Dataset Processing of Drivetrain Diagnostic Simulator

**Figure 16:**
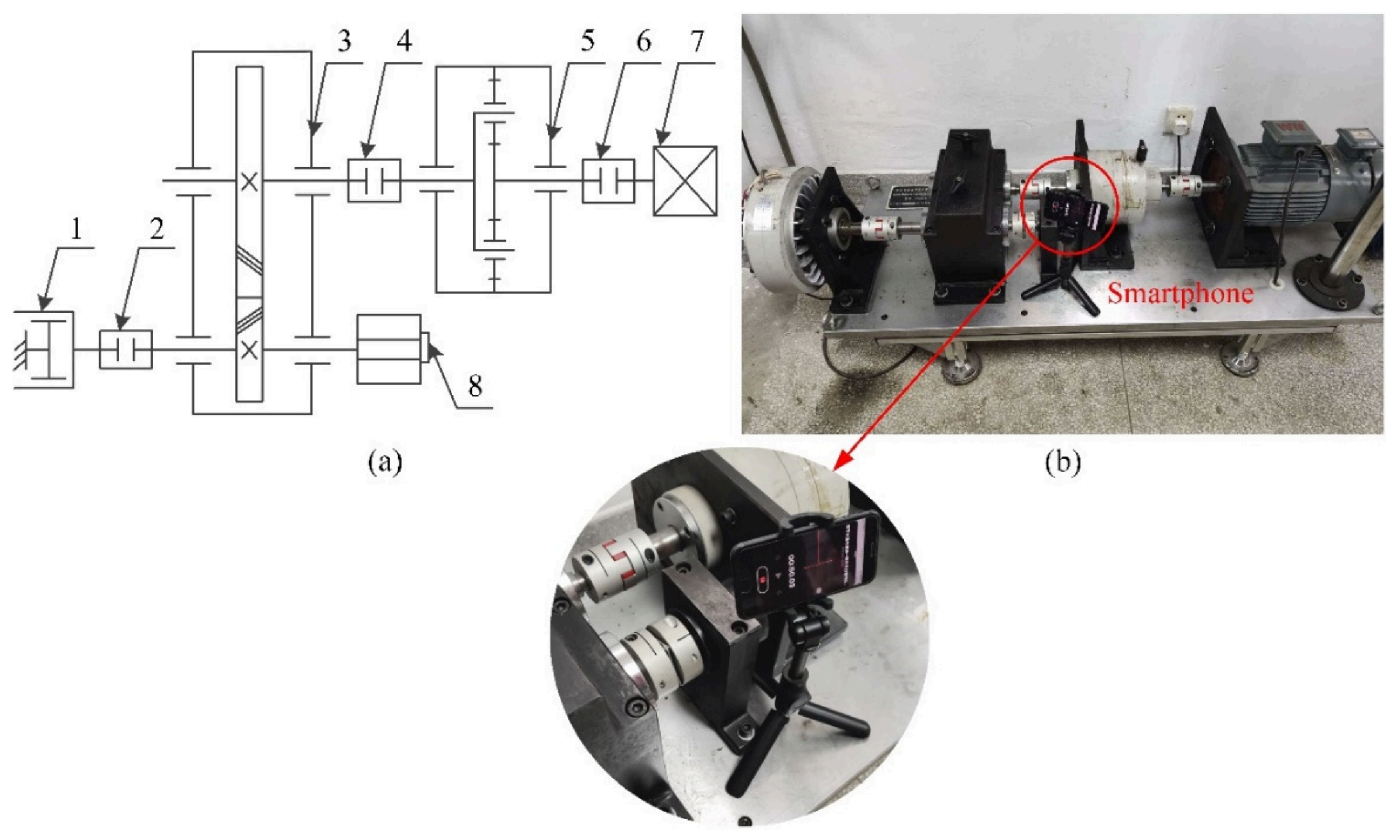
(**a**) The schematic diagram of the experimental apparatus, with 1—magnetic powder brake, 2, 4, 6—shaft coupling, 3—helical gear box, 5—planetary gear box, 7—variable motor, and 8—external bearing pedestal. (**b**) The experimental apparatus and signal acquisition equipment.

The vibro-acoustic signals of bearings were collected using the same apparatus as in Section 5.1. The smartphone was used to collect vibro-acoustic signals from four kinds of rolling bearings in the bearing pedestal, including normal bearings and bearings with inner race, ball, and outer race defects. The rotation speed of the motor was 1500 rpm. The sampling frequency of the smartphone was 48 kHz. As before, 100 samples with the length of 4096 points were collected. The schematic diagram and a picture of the apparatus with data acquisition equipment are shown in Figure 16. The parameters of the HRB-N205M bearing are shown in Table 1.

**Figure 17:**
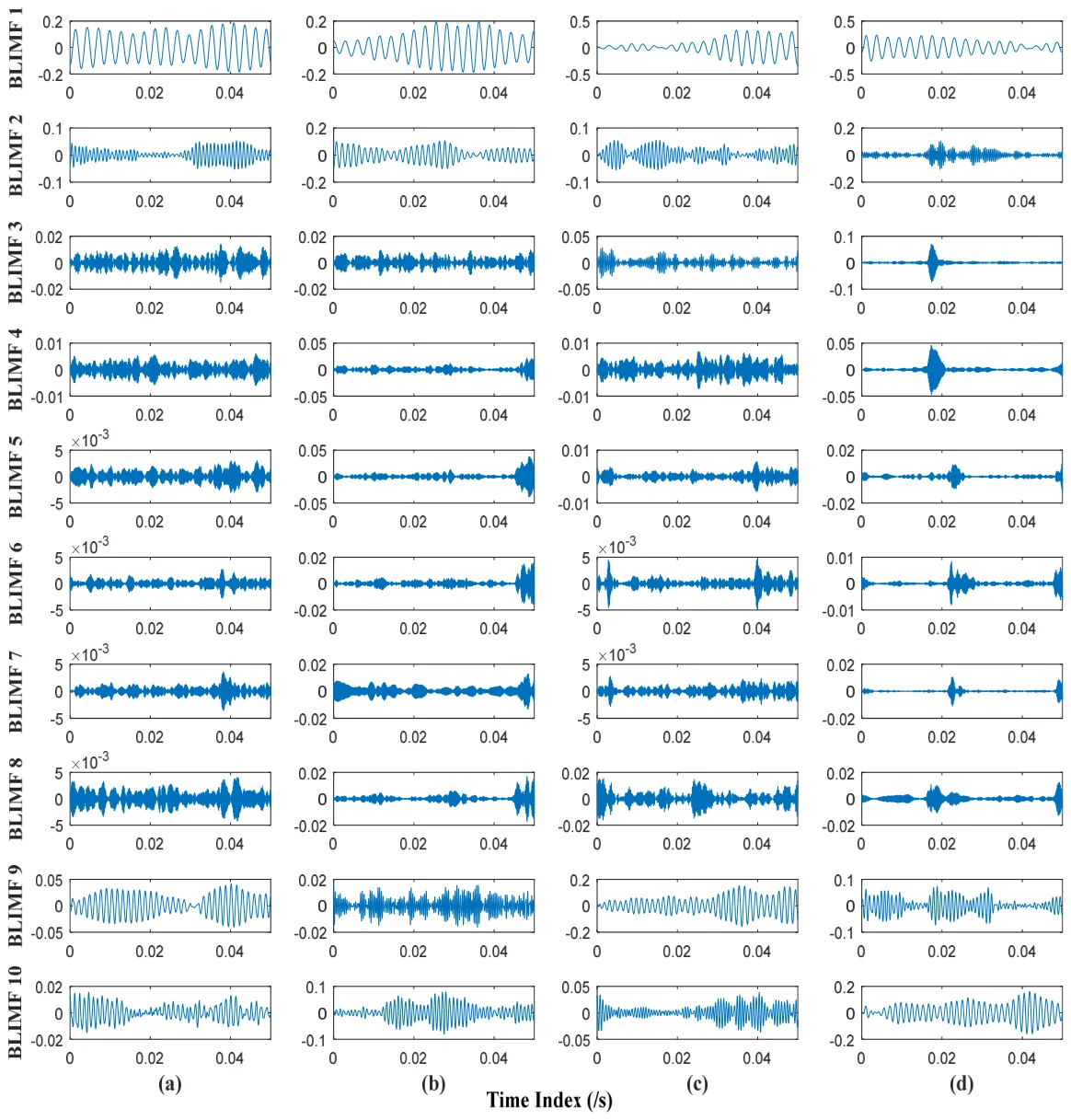
Time domain plots of the first 10 BLIMFs obtained by VMD of vibro-acoustic signals denoting different types of bearing: (**a**) normal, (**b**) inner race defect, (**c**) ball defect, and (**d**) outer race defect.

VMD was used to decompose the collected vibro-acoustic signals, and the algorithm’s parameters were determined using the same strategy in Section 5.1. They were determined using the Grasshopper optimization algorithm [35,60]. The mode order *K* and penalty factor *α* were set as 10 and 5000, respectively. The time domain plots of the 10 BLIMFs are shown in Figure 17a–d.

**Figure 18:**
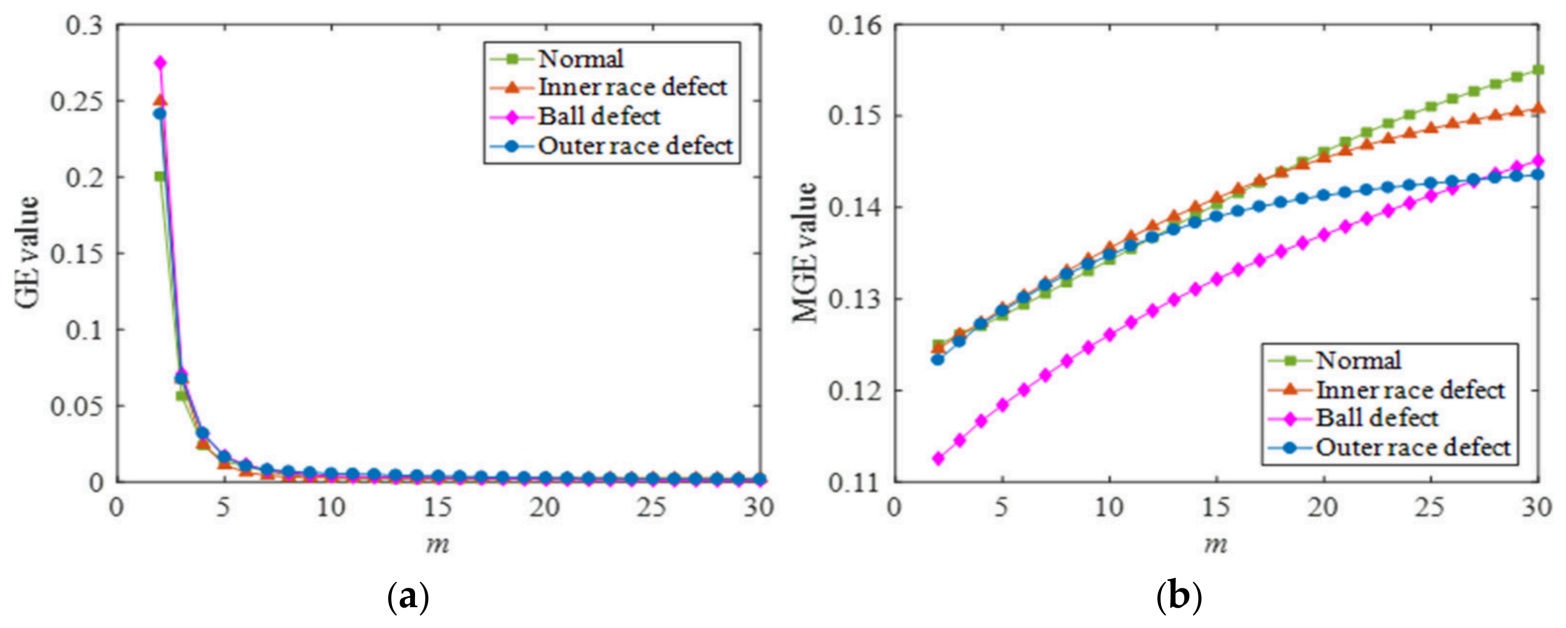
(**a**) GE and (**b**) MGE values, on multiple dimensions, of 7th BLIMF with the length of 4096 points.

**Figure 19:**
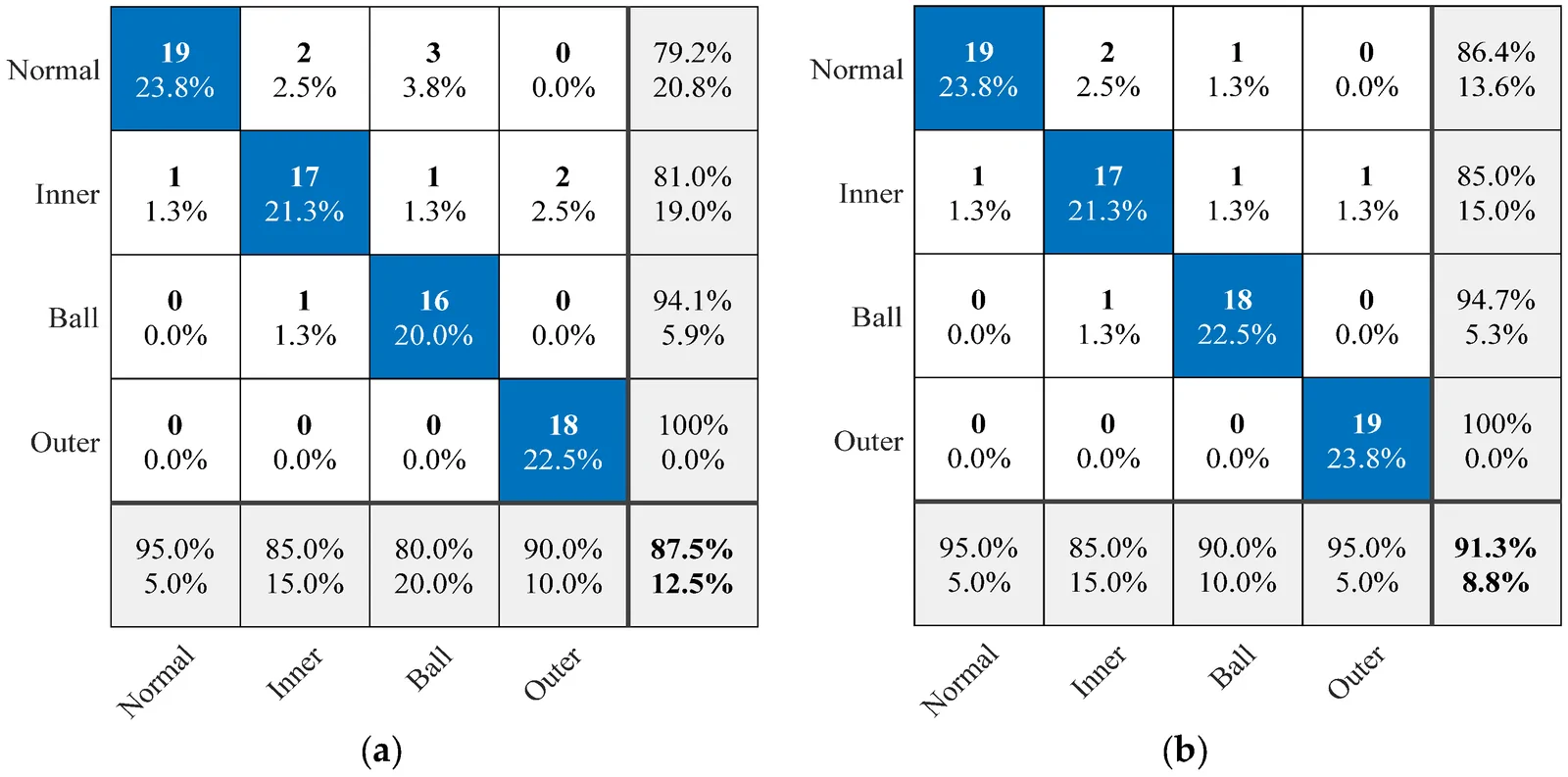

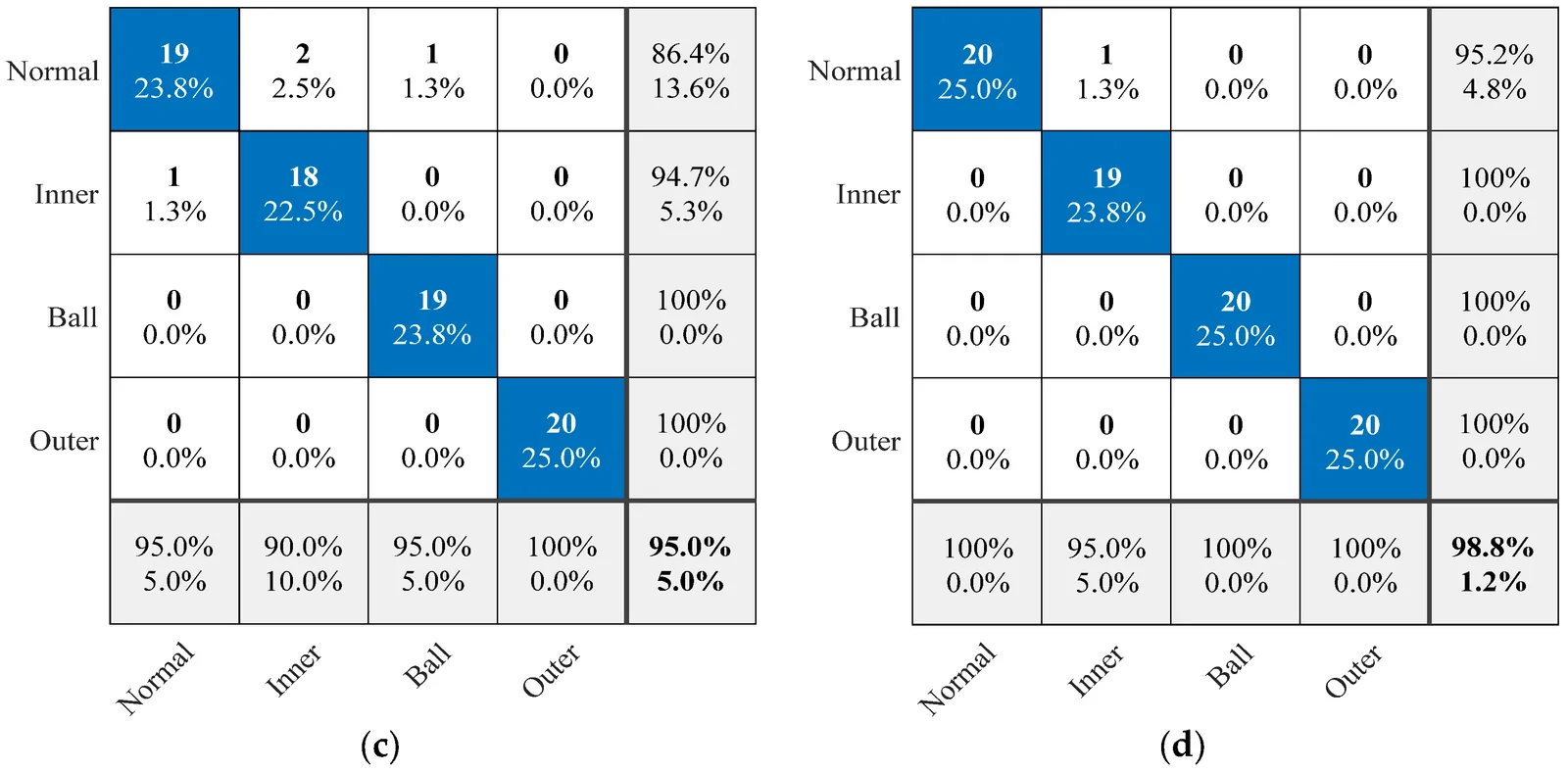
Confusion matrices of testing results: (**a**) EEMD and GE, (**b**) CEEMDAN and GE, (**c**) IMGE analysis using VMD and GE, and (**d**) IMGE analysis using VMD and MGE.

MGE was used to measure the complexity and regularity of BLIMFs. The dimension *m* was set at 30 to reflect the complexity and regularity of the vibro-acoustic signals. Figure 18 shows that GE values decrease monotonically for four types of bearing. The entropy values of the seventh BLIMF of four kinds of rolling bearings were mixed, and it is difficult to identify each bearing type from them, while the MGE values show significant differences. It can be concluded that the MGE values of BLIMFs characterize the dynamic properties of vibro-acoustic signals. The comparative analysis validates the effectiveness or improved performance of MGE in quantifying the complexity and regularity of vibro-acoustic signals from bearings. The 11–17-dimensional MGE values of the first seven BLIMFs obtained by VMD were calculated and used as the quantitative features of the vibro-acoustic signal. For 100 signal samples, all 7 × 7 feature matrices were obtained. Then, the feature matrices were put into the constructed CNN model. Deep learning is conducted by a convolutional layer, which has a 356-element two-dimensional filter, a max-pooling layer, and a two-element subsampling mask. The fully connected layer with 512 units was used to classify the learned features. In the output layer, the Softmax activation function was used, and in the other layers, the ReLU function was employed. The CNN was trained for 1000 epochs with minibatch gradient descent. For the training process, the categorical cross-entropy error and Adam optimizer was used, and the learning rate was 0.0001. For each bearing condition, 100 signal segments with a length of 4096 points were obtained. The segments were randomly divided into training, validation, and test sets with a ratio of 60:20:20. Because the recordings were collected under a controlled laboratory setup and the available segments share recording-session-dependent characteristics, this split is used to evaluate the within-dataset classification performance. The confusion matrices of the test results of different strategies are shown in Figure 19.

**Figure 20:**
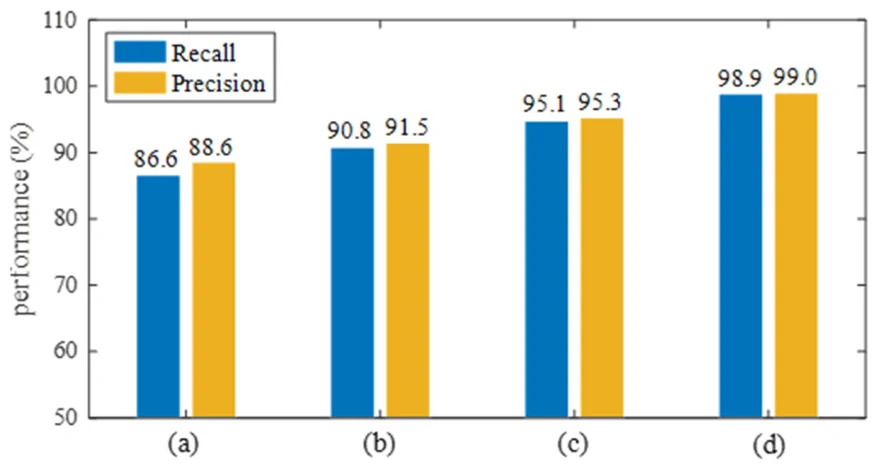
The average classification performance: (**a**) EEMD and GE, (**b**) CEEMDAN and GE, (**c**) IMGE analysis using VMD and GE, and (**d**) IMGE analysis using VMD and MGE.

Figure 19a shows that the classification accuracy of the CNN using EEMD and GE is 87.5%. The vibro-acoustic signals of rolling bearings are more complicated to analyze than those of gears. Figure 19b shows that the accuracy of CEEMDAN and GE is 91.3%. Figure 19c shows that the accuracy of IMGE analysis using VMD and GE is 95.0%. Figure 19d shows that the testing accuracy of CNN and IMGE analysis using VMD and MGE is 98.8%, validating the proposed multidimensional MGE. To accurately evaluate the performance of the proposed approach, the classification processes were both conducted 20 times. The average values of the recall and precision ratios are given in Figure 20.

Figure 20 shows that the recall values of the strategy using EEMD and GE, and CEEMDAN and GE, are 86.6% and 90.8%, respectively, while their precision values are 88.6% and 91.5%, respectively. This comparison validates the effectiveness of the proposed method. The quantitative features of the sub-signals are verified to depict the dynamic characteristics of the signals. Thus, they can be adopted as the input of the CNN classifier. The comparisons between the strategies of (a), (b), and (c) verify the effectiveness and improved performance of VMD in processing the vibro-acoustic signals of faulty rolling bearings. The recall and precision values of IMGE analysis using VMD and MGE were 98.2% and 99.0%, respectively. The consistency between the confusion matrix results and the high average recall and precision values obtained from 20 repeated experiments further indicate the stability of the proposed diagnostic method under the experimental conditions investigated in this study. The proposed approach achieves the optimal effect in classification of different types of rolling bearings. The application studies show that the proposed vibro-acoustic fault diagnosis method is feasible for use in rotary machinery.

## 6. Discussions and Conclusions

This paper proposes a novel vibro-acoustic fault diagnosis approach. It uses the strategy of intrinsic multidimensional Gnome Entropy (IMGE) analysis, consisting of variational mode decomposition (VMD) and the multidimensional mean Gnome entropy (MGE), to extract quantitative features of the vibro-acoustic signals. The quantitative features are used as the input of a convolutional neural network (CNN) to conduct deep learning, thus achieving accurate fault diagnosis. Rolling bearings and gears are typical rotating components, hence it can be inferred that the proposed approach can also be used to process the vibro-acoustic signals of rotary machinery. The major findings of the paper are summarized as follows:

(1) The processing of vibro-acoustic signals collected by a microphone or a smartphone is useful for diagnosing faults in rotary machinery. The quantitative features extracted from vibro-acoustic signals can be adopted as effective features for further analysis.
(2) As an optimized algorithm of EMD, VMD can better decompose vibro-acoustic signals into a set of BLIMFs. BLIMFs can accurately characterize the dynamic behavior of signals and possess specific sparsity and filter-bank properties. VMD performs well in representing the dynamic characteristics of vibro-acoustic signals.
(3) The proposed MGE employs mean processing during the GE calculation procedure. This step improves the accuracy and stability of GE values in multiple dimensions. The comparative analysis using different datasets verifies the effectiveness and improved performance of MGE. Its effectiveness in processing vibro-acoustic signals has been validated.
(4) In this study, CNN was used to perform high-level reasoning based on the extracted feature matrices. The CNN-based deep-learning method takes advantage of the quantitative features of vibro-acoustic signals and accurately classifies faults in rotary machinery.

The effectiveness of the proposed vibro-acoustic fault diagnosis framework was evaluated under specific conditions. The results show that the proposed vibro-acoustic approach is promising in the field of fault diagnosis of rotary machinery. MGE was mainly evaluated from the perspectives of numerical stability and fault-class separability. These criteria do not constitute a complete validation of the physical accuracy of MGE as a universal complexity measure. Future work will collect independent vibro-acoustic recordings under multiple rotational speeds and loads. Cross-condition experiments will then be conducted by training the model using selected operating conditions and testing it directly on previously unseen speeds and loads, without redistributing segments from the same recording condition between the training and test sets.

## Figures and Tables

**Table 1:** The parameters of the HRB-N205M rolling bearing.

Ball Number	Contact Angle	Ball Diameter	Outside Diameter	Inside Diameter	Ball Number
13	0	15	52	25	13

## Data Availability

The data that support the findings of this study are available from the corresponding author upon reasonable request.
